# GM-CSF Priming Drives Bone Marrow-Derived Macrophages to a Pro-Inflammatory Pattern and Downmodulates PGE_2_ in Response to TLR2 Ligands

**DOI:** 10.1371/journal.pone.0040523

**Published:** 2012-07-13

**Authors:** Carlos Arterio Sorgi, Stephanie Rose, Nathalie Court, Daniela Carlos, Francisco Wanderley Garcia Paula-Silva, Patricia Aparecida Assis, Fabiani Gai Frantz, Bernhard Ryffel, Valerie Quesniaux, Lúcia Helena Faccioli

**Affiliations:** 1 Departamento de Análises Clínicas, Toxicológicas e Bromatológicas, Faculdade de Ciências Farmacêuticas de Ribeirão Preto – Universidade de São Paulo – Ribeirão Preto, SP – Brazil; 2 CNRS, UMR6218, Orleans, France; 3 Orleans University, Molecular Immunology and Embryology, Orleans, France; University of São Paulo, Brazil

## Abstract

In response to pathogen recognition by Toll-like receptors (TLRs) on their cell surface, macrophages release lipid mediators and cytokines that are widely distributed throughout the body and play essential roles in host responses. Granulocyte macrophage colony-stimulating factor (GM-CSF) is important for the immune response during infections to improve the clearance of microorganisms. In this study, we examined the release of mediators in response to TLR2 ligands by bone marrow-derived macrophages (BMDMs) primed with GM-CSF. We demonstrated that when stimulated with TLR2 ligands, non-primed BMDMs preferentially produced PGE_2_ in greater amounts than LTB_4_. However, GM-CSF priming shifted the release of lipid mediators by BMDMs, resulting in a significant decrease of PGE_2_ production in response to the same stimuli. The decrease of PGE_2_ production from primed BMDMs was accompanied by a decrease in PGE-synthase mRNA expression and an increase in TNF-α and nitric oxide (NO) production. Moreover, some GM-CSF effects were potentiated by the addition of IFN-γ. Using a variety of TLR2 ligands, we established that PGE_2_ release by GM-CSF-primed BMDMs was dependent on TLR2 co-receptors (TLR1, TLR6), CD14, MyD88 and the nuclear translocation of NFκB but was not dependent on peroxisome proliferator-activated receptor-γ (PPAR-γ) activation. Indeed, GM-CSF priming enhanced TLR2, TLR4 and MyD88 mRNA expression and phospho-IκBα formation. These findings demonstrate that GM-CSF drives BMDMs to present a profile relevant to the host during infections.

## Introduction

The innate immune response is initiated by microbe recognition, phagocytosis and activation of numerous cells; macrophages seem to be at the focal point of this scenario. Through the production of inflammatory mediators macrophages exert a broad variety of functions [1]. Murine macrophages have been used as an important primary cell model for the investigation of the release and function of pro-inflammatory mediators. Because macrophages are derived from bone marrow cells, this seems a valuable tool to obtain high numbers of cells from a single mouse [2]. Transgenic and gene-disrupted mice allow the study of particular mechanisms, which has emphasized the need for cultures of primary cells, like as BMDM [2]. The specific profile of metabolites produced by phagocytes depends on the stage of differentiation, anatomic site of residence [3], and mainly depends on the activation pathway. The surface expression of receptors and co-receptors, such as toll-like receptors (TLR), are modulated by external factors, including cytokines and chemokines, produced in the microenvironment. The activation of TLR2 pathways by bacterial and yeast cell wall components is enhanced by GM-CSF [4]. GM-CSF is also described as a factor for granulocytes priming for leukotrienes (LTs) synthesis subsequent to stimulation with chemotactic factors [5] **.** The increased release of LT results from GM-CSF priming through increase of arachidonic acid (AA) release**,** enhancement of 5-Lipoxygenase (5-LO) activation [6], [7] and rapid augmentation of mRNA translation to increase 5-LO protein levels [8]. Likewise, when alveolar macrophages (AM) are stimulated with GM-CSF, the production of LT is increased [9], validating its role as a primary stimulator of different cell populations.

GM-CSF is an immune regulatory cytokine produced by macrophages, endothelial cells, alveolar epithelial cells and T cells in response to pro-inflammatory cytokines, activating a variety of cells, such as dendritic cells, neutrophils and macrophages [10]. This cytokine is responsible for the survival, proliferation, differentiation and function of myeloid cells [11]. The clearance of microorganisms from the lungs of GM-CSF-deficient mice is extremely affected [12], [13], providing evidence of its importance in the immune response during infectious diseases. AMs phagocyte a range of pathogens and particles through mechanisms mediated by receptor interactions that can be regulated by GM-CSF [13], [14].

Microbes and their components activate innate immune responses through interaction of their PAMPs with TLRs. TLR2 recognizes bacterial components, such as peptidoglycan (PGN), bacterial triacylated lipoprotein (Pam_3_CSK_4_), mycoplasma diacylated lipoprotein (Malp2), lipoarabinomannan (AraLAM), zymosan and protozoan GPI anchors [15], [16], [17], [18], [19], [20]. Furthermore, TLR4 is essential for responses to LPS [21]. TLR activation culminates in the production of cytokines, chemokines, and other pro-inflammatory molecules to evoke host defense responses and initiate acquired immunity [22].

Lipid mediators, such as LT and prostaglandins (PG), are widely recognized for their pro-inflammatory activities [23], [24]. PGE_2_ enhances vasodilation, edema formation and vascular permeability [24]. LTB_4_ is a potent chemotactic and chemokinetic mediator and acts as a leukocyte activator [25]. The role of lipid mediators during infections has been shown. LT can enhance the phagocytic activity of neutrophils and macrophages and increase their capacity to kill microbes and produce antimicrobial mediators [25]. Moreover, *Mycobacterium tuberculosis*-infected mice treated with 5-LO pathway inhibitor MK886 have an increased susceptibility to the disease [26]. This inhibition also results in exacerbation of pulmonary histoplasmosis [27]. The role of PG in host defense is also recognized. PGI_2_ regulates the killing and phagocytosis of *Escherichia coli* by alveolar and peritoneal macrophages [28]. Treatment with COX-1/−2 inhibitors favor parasitism by *Strongyloides venezuelensis* due to a shift in the immune response [29] and liberation of PGE_2_ after ingestion of apoptotic cells by phagocytes results in poor clearance of *Streptococcus pneumoniae* [30].

However, in contrast to the wealth of data exploring the TLR-dependent cytokine expression and activation of oxidant production, the mechanisms governing the TLR-mediated activation of lipid mediators production in primary macrophages is unclear. The production of eicosanoids and the macrophage differentiation/maturation process play an important role in innate immunity. Here, we address the effect of a set of TLR2-ligands on the production of PGE_2_ and LTB_4_ by GM-CSF primed BMDM and the correlation with other pro-inflammatory mediators, such as TNF-α and Nitric Oxide (NO).

## Methods

### Mice

Six- to 8-week old C57Bl/6 mice deficient for TLR2 [17], TLR1 [31], TLR6 [32], CD14 [33], MyD88 [34] and TLR4 [35] and their strain-matched wildtype (WT) controls from both sexes were bred under specific pathogen-free conditions in the Transgenose Institute animal breeding facility (UPS44, CNRS, Orleans, France). This study was carried out in strict accordance with the recommendations in the Guide for the Care and Use of Laboratory Animals of the Regional ethics committee (Permit Number: CL2008-011). All surgery was performed under CO_2_/O_2_ excess atmosphere, and all efforts were made to minimize suffering.

### Priming and Stimulation of Primary Macrophage Cultures in vitro

Murine bone marrow cells were isolated from femurs and cultivated (10^6^/ml) for 7 days (37°C and 5% CO_2_) in Dulbecco’s minimal essential medium (DMEM) supplemented with 10 mM L-glutamine (Sigma), 100 U/mL penicillin (Invitrogen) and 100 U/mL streptomycin (Invitrogen), 20% horse serum and 30% L929 cell-conditioned medium as a source of macrophage colony-stimulating factor (M-CSF). After suspending in cold phosphate-buffered saline (PBS), washing, and re-culturing for 3 days in fresh medium, the cell preparation contained a homogenous population of macrophages (97–98% CD11b^+^F4/80^+^). The bone marrow derived macrophages (BMDM) were plated in 48-well microculture plates (Nunc) at a density of 5×10^5^ cells/well in DMEM supplemented with 10 mM L-glutamine, 100 U/mL penicillin and 100 U/mL streptomycin and10% fetal bovine serum (FBS) and cultured at 37°C in a humidified atmosphere of 5% CO_2_ for 18 h. BMDM were primed (or not) with 10% J558L cell-conditioned medium as a source of granulocyte macrophage colony-stimulating factor (GM-CSF) (3,5 ng/10^5^ cells) for 24 h before stimulation or in a combination with interferon-γ (IFN-γ) (500 UI/ml). Subsequently, BMDM were stimulated with increased concentrations of TLR-ligands as AraLAM-MS (lipoarabinomannan from *Mycobacterium smegmatis)* (InvivoGen) (5–5000 ng/mL), LM-MS (lipomannan from *M. smegmatis)* (InvivoGen) (5–5000 ng/mL), Malp2 (*S*-(2,3-bisacyloxypropyl)-cysteine-GNNDESNISFKEK) (Alexis Biochemicals) (0,3–300 ng/ml), synthetic bacterial lipopeptide Pam_3_-CSK_4_ ((*S*)-2, 3-bis(palmitoyloxy)-(2-*RS*)-propyl]-*N*-palmitoyl-(*R*)-Cys-(*S*)-Ser-Lys4-OH)tri-hydrochloride, EMC Microcollections) (5–5000 ng/ml) or LPS (*Escherichia coli*, serotype O111:B4; InvivoGen) (50–1000 ng/ml) diluted in DMEM for 24 h at 37°C in a CO_2_ atmosphere. To determine the time of stimulation, we performed a time-response titration experiment and chose 24 h of stimulation based on a maximal production of TNF-α and NO (data not shown). Medium (DMEM) was used as negative control. Also, to neutralize potential endotoxin presence in the stimulus solutions, they were pretreated with Polymyxin-B (Sigma) at 10 µg/mL for 20 min and then added to the cell culture. In other experiments, to investigate the participation of peroxisome proliferator-activated receptor-γ PPAR-γ, BMDM were treated for 30 min before stimulation with an antagonist of PPAR-γ (GW9662) (Sigma) at 1 µM at 37°C and stimulated with the TLR-ligands above or infected with BCG (MOI, 1∶1) or heat-killed *M. tuberculosis* H37Rv (HK H37) (MOI, 1∶1) during 24 h. Vehicle (DMSO 0.01% in DMEM) was used as control. The culture supernatants were harvested and analyzed immediately or stored at 20°C until further use. The absence of cytotoxicity of the stimuli was controlled using the incorporation of MTT (3-(4,5-dimethylthiazol-2-yl)-2,5-diphenyltetrazolium bromide) (Sigma).

### Cytokine Enzyme-Linked Immunosorbent Assay

Supernatants were harvested and assayed for cytokine content using the commercially available enzyme-linked immunosorbent assay reagents for TNF-α and IL-10 (Duoset R&D). The detection limit for both cytokines was 7 pg/mL.

### Nitrite Measurements

Nitrite concentrations in the cell supernatants were measured using the Griess reaction (3% phosphoric acid, 1% *p*-aminobenzene sulfonamide, 1% *N*-1-napthyl ethylenediamide) as previously described [36].

### Eicosanoids Measurements

LTB_4_ and PGE_2_ concentrations were measured directly in cell-free supernatant from primary macrophage culture. Eicosanoids were assayed using enzyme-linked immunoassays according to the manufacturer’s instructions (Cayman Chemical).

#### Analysis of gene expression by real-time polymerase chain reaction (qRT-PCR)

Expression of mRNA in BMDM cells after treatment with GM-CSF for 4, 8 and 18 h was evaluated using a custom RT_2_ Profiler PCR Array kit (Qiagen). Total RNA from BMDM cells was isolated using the RNeasy Mini kit (Qiagen), and the reverse transcription of 3 µg of RNA was performed using the RT_2_ HT First Strand kit (Qiagen). Each cDNA sample was processed in 96-well plates containing sets of pre-defined genes (*Tlr1, Tlr6, Tlr4, Tlr2, Cd14, Itgb2, Ticam1, Ticam2, Alox5, Alox5ap, Ptgs2 and Ptges2*), including *Actb* and *Gapdh* as endogenous internal controls in all sets. Amplification was performed duplicates in an Eppendorf Mastercycler ep realplex 4 (Eppendorf). The 2–ΔΔCt method was used in the analysis of the RT-PCR data.

### Measurement of Total and Phosphorylated Proteins by ELISA

Following priming and LM (5000 ng/mL) stimulation, 3×10^6^ cells were lysed in cell lysis buffer (Cell Signaling Technology). Cell lysates were analyzed, and proteins were semi-quantified using the PathScan® total-NFκBp65, phospho-NFκBp65 (Ser536) and total-IκBα, phospho-IκBα (Ser32) sandwich ELISA kits, according to the manufacturer’s instructions (Cell Signaling Technology). Brieﬂy, the lysates were diluted (10 µg/mL), and 100 µL of lysate was added to wells pre-coated with primary antibody. The plate was incubated overnight at 4°C and then washed four times. After washing the plate, the detection antibody was added to the wells, and the plate was incubated for 1 h at 37°C. After a second washing process, horseradish peroxidase (HRP)-linked secondary antibody was added, and the plate was incubated for 30 min at 37°C. Substrate was added, and the plate was incubated for 30 min at room temperature. Finally, the reaction was stopped, and the samples were read at an absorbance of 450 ηm. The results were expressed as the percentage of relative expression (absorbance) of control *versus* that produced by primed BMDMs stimulated with LM.

### Nuclear Translocation of NFκB

BMDM were adhered to cover slides and cultured for 18 h at 37°C in a humidified atmosphere of 5% CO_2_. Cells were primed with GM-CSF for 24 h and subsequently stimulated with TLR2-ligands as described above for 2 h at 37°C and 5% CO_2_. Next, the monolayer was washed by gentle agitation with PBS, fixed in 4% paraformaldehyde, permeabilized with Triton X-100 (0.5%) and used for immunolocalization of NFκB as described [37], with minor modifications. BMDM were incubated with goat anti-murine NFκBp65 antibody (Santa Cruz Biotechnology) for 1 h at room temperature, washed, and incubated with swine anti-goat IgG FITC Ab (Sigma). Cells presenting NFκB nuclear translocation were scored by confocal microscopy (Leica SP2).

### Statistical Analysis

Results were expressed as mean ± SEM. Statistical significance was determined with Graph Pad Prism software (Version 4.0, San Diego, CA) by unpaired t-test analysis of variance. Values of *p*<0.05 were considered statistically significant.

## Results

### 1. GM-CSF Priming Reduces Macrophage Production of PGE_2_

**Figure 1:**
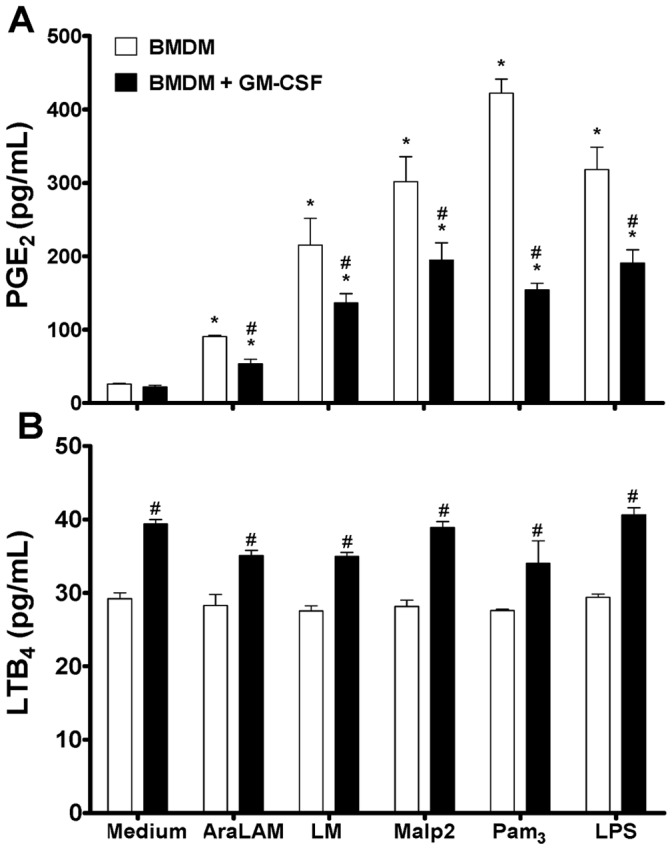
GM-CSF-modified lipid mediators released by BMDM in response to bacterial TLRs-ligands. BMDM from WT mice were primed (or not) with GM-CSF for 24 h and then incubated with AraLAM (5000 ng/mL), LM (5000 ng/mL), Malp2 (300 ng/mL), Pam_3_-CSK_4_ (5000 ng/mL) or LPS (500 ng/mL) for 24 h. PGE_2_ (**A**) and LTB_4_ (**B**) release were measured in the supernatant by ELISA. Medium alone was used as a negative control. Results are the average ± S.E.M from mice cells (*n* = 6 per group), from two independent experiments; *, *p*<*0.05* compared with unstimulated cells; **^#^**, *p<0.05* compared with non-priming GM-CSF cells.

We characterized the capacity of different TLR2 ligands derived from bacteria to induce the release of PGE_2_ and LTB_4_ from macrophage after priming with GM-CSF. Cells were primed, or not primed, with GM-CSF and then stimulated with the TLR-agonists AraLAM, LM, Malp2, Pam_3_-CSK_4_ or LPS TLR4 ligand as a positive control. As shown in Figure 1A, unprimed macrophage stimulated with AraLAM, LM, Malp2, Pam_3_-CSK_4_ and LPS produced high levels of PGE_2_ as compared with the negative control; however, the response to AraLAM stimulation was slightly weaker. GM-CSF priming resulted in a significant decrease in PGE_2_ production by stimulated macrophages. The kinetics of stimulation was similar to all ligands, with the maximum response obtained after 24 h of incubation (data not shown). Macrophage priming with GM-CSF resulted in a small increase in LTB_4_ release as compared with unprimed cells (Fig. 1B). However, GM-CSF priming did not affect TLRs induced LTB_4_ production (Fig. 1B). Similarly, no effect of GM-CSF priming was observed on LTC_4_ release after TLR-agonist stimulation in the same conditions (data not shown). Thus, GM-CSF priming decreased PGE_2_ release by TLR-activated macrophages.

### 2. GM-CSF Priming Enhances TLR2 Ligands-induced TNF-α and NO Release

**Figure 2:**
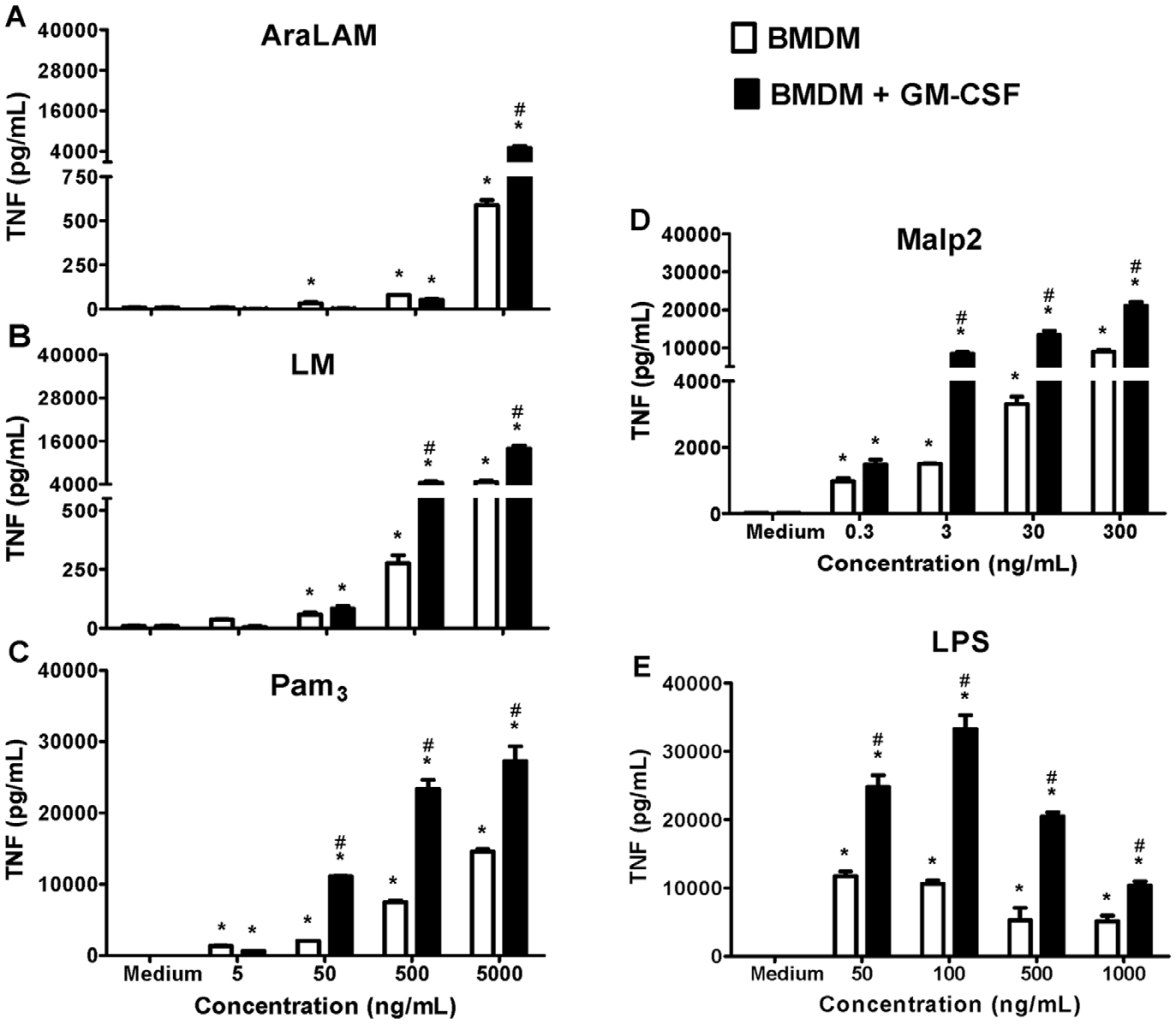
Effect of GM-CSF priming on TNF-α secretion by BMDM. BMDM from WT mice were primed (or not) with GM-CSF for 24 h and then incubated for 24 h with increasing concentrations of AraLAM (5–5000 ng/mL) (**A**), LM (5–5000 ng/mL) (**B**), Pam_3_-CSK_4_ (5–5000 ng/mL) (**C**), Malp2 (0.3–300 ng/mL) (**D**) or LPS (50–1000 ng/mL) (**E**). Medium alone was used as a negative control. Supernatants were harvested, and ELISA was used to determine the cytokine content. Results are the average ± S.E.M from mice cells (*n* = 6 per group), from two independent experiments; *, *p*<*0.05* compared with unstimulated cells; **^#^**, *p<0.05* compared with non priming GM-CSF cells.

The effects of GM-CSF priming on macrophage release of TNF-α and NO in response to TLR2 or TLR4 stimulation were further analyzed. Macrophages were treated with increasing concentrations of TLR-ligands for 24 h. A time-dependent increase in TNF-α and NO, which peaked after incubation for 24 h, was observed (data not shown). Therefore, in all subsequent experiments, TNF-α and NO was measured at this time point. We also evaluated the concentration-dependent effect of TLR-ligands and observed that Malp2 (Fig. 2D), LM (Fig. 2B) and Pam_3_-CSK_4_ (Fig. 2C) induced maximal responses at 300 ng/ml, 5000 ng/mL and 5000 ng/mL, respectively. Priming with GM-CSF induced an increase of the peak TNF-α release, obtained at these concentrations. Moreover, AraLAM induced significant concentrations of TNF-α only at 5000 ng/mL (Fig. 2A). GM-CSF priming also greatly increased LPS-induced TNF-α release (Fig. 2E).

**Figure 3:**
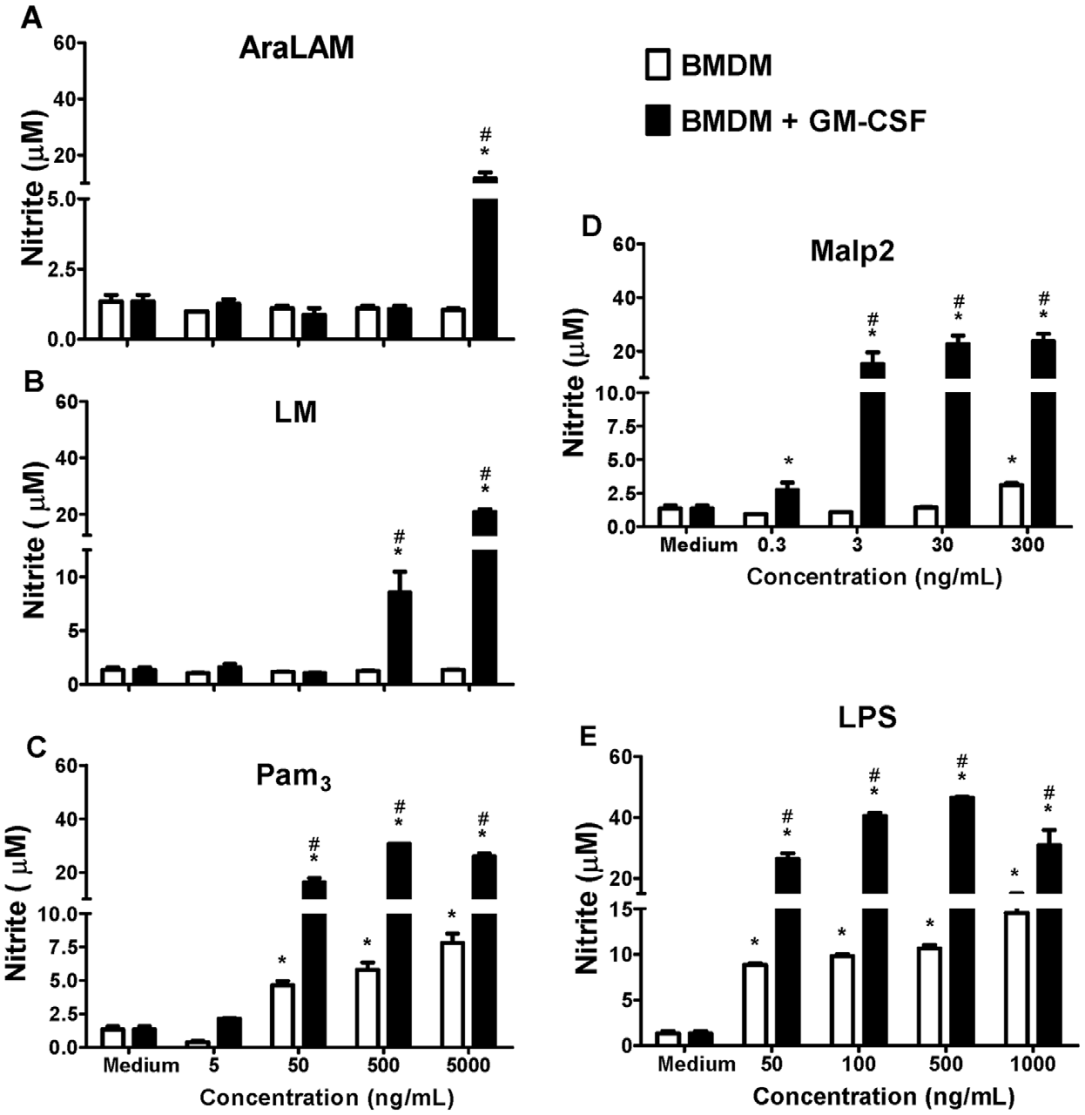
Effect of GM-CSF priming on NO production by BMDM. BMDM from WT mice were primed (or not) with GM-CSF for 24 h and then incubated for 24 h with increasing concentrations of AraLAM (5–5000 ng/mL) (**A**), LM (5–5000 ng/mL) (**B**), Pam_3_-CSK_4_ (5–5000 ng/mL) (**C**), Malp2 (0.3–300 ng/mL) (**D**) or LPS (50–1000 ng/mL) (**E**). Medium alone was used as a negative control. Supernatants were harvested, and the Nitrite content determined by the Griess Method. Results are the average ± S.E.M from mice cells (*n* = 6 per group), from two independent experiments; *, *p*<0.05 compared with unstimulated cells; **^#^**, *p<0.05* compared with unprimed GM-CSF cells.

AraLAM (Fig. 3A), LM (Fig. 3B) and Malp2 (Fig. 3D) induced no or only a weak production of NO in unprimed cells, but NO release was increased synergistically after GM-CSF priming. In addition, LPS (Fig. 3E) and Pam_3_-CSK_4_ (Fig. 3C) stimulated NO release dose-dependently and a significant enhancement in NO release was also observed after GM-CSF priming.

The capacity of TLR2 to recognize a vast array of compounds has been attributed to the ability of TLR2 to heterodimerize with other TLR co- and accessory receptors, which defines their ligand specificity [38]. The TLR2 co-receptors include TLR1 and TLR6. In addition, recognition of TLR2 agonists is influenced by several accessory receptors, including CD14 [39]. Most TLRs, including TLR2, recruit the MyD88 adaptor molecule, which leads to the induction of downstream signaling events through activation of nuclear factor NFκB and mitogen-activated protein (MAP) kinases [40], [41].

To further identify whether TLR1, TLR6, CD14 and MyD88 are involved in the signaling pathway of NO and TNF-α production by GM-CSF primed macrophages, we analyzed the TLR pathway dependence in macrophages from gene-deficient mice. Low concentrations of TNF-α (Table 1) were produced by GM-CSF-primed TLR2^−/−^ and CD14^−/−^ macrophages after stimulation with AraLAM and LM. In contrast, after Malp2 and Pam_3_-CSK_4_ stimulation, TNF-α was efficiently released by GM-CSF-primed CD14^−/−^ but not by TLR2^−/−^ macrophages. In the absence of either TLR1 or TLR6, there was a partial decrease in TNF-α release following Pam_3_-CSK_4_ stimulation, but TNF-α release was totally abolished in GM-CSF-primed TLR6^−/−^ macrophages stimulated with Malp2. As expected LPS-induced TNF-α release was essentially absent in GM-CSF-primed TLR4^−/−^ or CD14^−/−^ macrophages. TNF-α production was strongly impaired or not detected in the supernatants of MyD88^−/−^ macrophages in the presence of all stimuli used (Table 1). Similarly, the induction of NO (Table 2) by AraLAM, LM, Malp2 and Pam_3_-CSK_4_ was strongly dependent on TLR2 and MyD88. In addition, NO release by GM-CSF-primed macrophages stimulated with LM was dependent on CD14 but dependent on TLR6 when stimulated with Malp2. As described, LPS stimulation was totally dependent on CD14 and TLR4 and partially dependent on MyD88 (Table 2). Therefore, GM-CSF priming did not alter the TLR pathway usage for macrophage TNF and NO release.

**Table 1:** Effect of GM-CSF priming on TNF-α production by BMDM in response to bacterial stimulus^a^.

Stimulus	TLR2^−/−^	TLR1^−/−^	TLR6^−/−^	TLR4^−/−^	CD14^−/−^	MyD88^−/−^
**AraLAM**	99.3±0.4^b^	1.7±10.2^c^	−4.4±12.3^c^	33.6±12.5	76.6±2.2^b^	99.5±0.1^b^
**LM**	89.9±2.9^b^	−1.6±0.2^c^	−7.1±4.8^c^	36.8±5.0^b^	69.3±1.5^b^	92.9±0.6^b^
**Malp2**	99.7±0.3^b^	15.8±1.5	99.1±0.5^b^	13.4±1.8	11.2±23.6	99.7±0.1^b^
**Pam_3_**	99.0±1.2^b^	44.0±3.2^b^	35.0±0.2^b^	15.9±0.7	−0.8±3.8^c^	99.7±0.2^b^
**LPS**	−21.3±17.4^c^	−19.7±14.5^c^	−19.5±1.7^c^	99.7±0.7^b^	79.1±1.3^b^	94.3±0.1^b^

a BMDM from WT (C57Bl/6) or deficient mice (^−/−^) for TLR2, TLR1, TLR6, TLR4, CD14 and MyD88 primed with GM-CSF, were incubated with AraLAM (5000 ng/mL), LM (5000 ng/mL), Malp2 (300 ng/mL), Pam_3_-CSK_4_ (5000 ng/mL) or LPS (500 ng/mL) for 24 h *in vitro*. TNF-α was measured by ELISA. Percentage of inhibition of TNF-α production in deficient mice compared to WT.

b *p<0.05*, significantly *versus* WT production. Data are mean ± S.D, *n* = 6 mice per genotype from two independent experiments.

c Negative values corresponds an increment in correlation with WT production.

### 3. PGE_2_ Induced by TLR2 Ligands Depend on Co-receptors and MyD88

**Figure 4:**
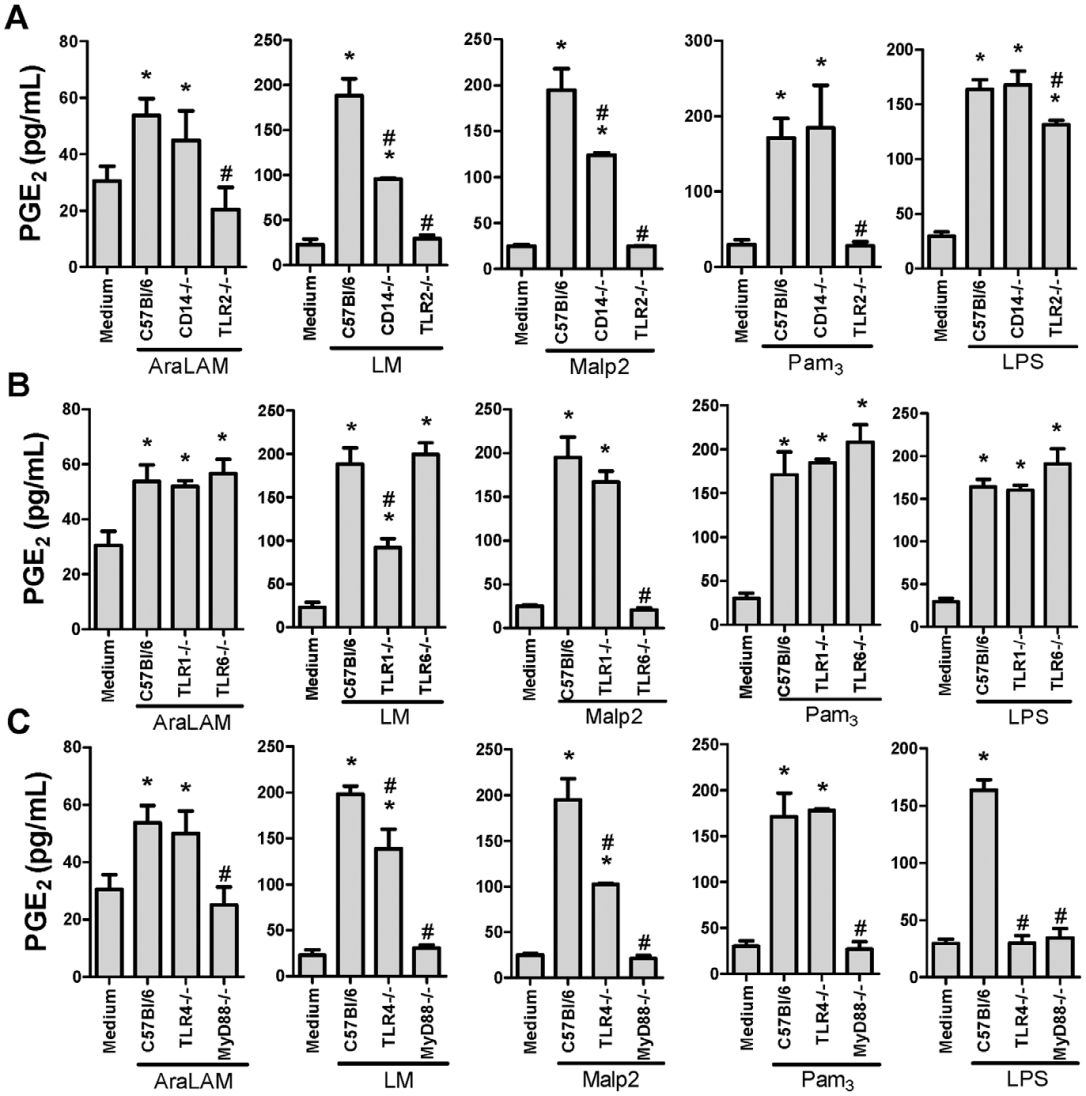
TLRs, co-receptors and MyD88 contribute to PGE_2_ production by GM-CSF primed BMDM. BMDM from WT (C57Bl/6) or mice deficient for TLR2 or CD14 (**A**), TLR1 or TLR6 (**B**), or TLR4 or MyD88 (**C**) were primed with GM-CSF for 24 h and then incubated for 24 h with AraLAM (5000 ng/mL), LM (5000 ng/mL), Malp2 (300 ng/mL), Pam_3_-CSK_4_ (5000 ng/mL) or LPS (500 ng/mL) *in vitro*. Medium alone was used as negative control. ELISA was used to measure the PGE_2_ production in the supernatants. Results are the average ± S.E.M (*n* = 6 per genotype), from two independent experiments; *, *p*<0.05 compared with unstimulated cells; **^#^**, *p<0.05* compared with WT mice.

The participation of TLR pathways in the production of PGE_2_ by GM-CSF-primed macrophages was then evaluated in macrophages from gene deficient mice. After stimulation with AraLAM, a significant increase in PGE_2_ production was observed in GM-CSF-primed macrophages cultures from C57Bl/6, CD14^−/−^, TLR1^−/−^, TLR6^−/−^ and TLR4^−/−^ mice, while this effect was abrogated in GM-CSF-primed, TLR2^−/−^ macrophages (Fig. 4A). Furthermore, LM stimulated PGE_2_ release from WT macrophages, but the absence of TLR2 dramatically reduced this effect (Fig. 4A). Macrophages deficient for TLR6 responded to LM as efficiently as WT cells, but the production of PGE_2_ was reduced in TLR1^−/−^ (Fig. 4B) and CD14^−/−^ (Fig. 4A) macrophages. MalP-2 induction of PGE_2_ was reduced in GM-CSF primed, TLR2^−/−^, CD14^−/−^ (Fig. 4A), TLR6^−/−^ (Fig. 4B) or TLR4^−/−^ (Fig. 4C) macrophages. GM-CSF primed, TLR2^−/−^ macrophages showed a severely impaired response to Pam_3_-CSK_4_ stimulation (Fig. 4A) while no significant difference in PGE_2_ production was observed in TLR1^−/−^ or TLR6^−/−^ macrophages (Fig. 4B). GM-CSF primed, TLR4^−/−^ cells showed an impaired production of PGE_2_ when stimulated with LPS (Fig. 4C), as expected. All of the TLRs evaluated in this work signal through MyD88, and our results clearly indicate the participation of this adaptor protein in the production of PGE_2_ after GM-CSF priming (Fig. 4C). Therefore, macrophage GM-CSF priming did not alter TLR pathway requirement for PGE_2_ release.

**Table 2:** Effect of GM-CSF priming on NO production by BMDM in response to bacterial stimulus^a^.

Stimulus	TLR2^−/−^	TLR1^−/−^	TLR6^−/−^	TLR4^−/−^	CD14^−/−^	MyD88^−/−^
**AraLAM**	55.6±6.9^b^	−10.0±30.3^c^	12.4±27.9	35.2±6.8^b^	34.2±39.5	98.4±2.1^b^
**LM**	79.0±10.1^b^	−25.8±23.7^c^	5.8±22.5	17.3±5.3	67.3±2.1^b^	83.0±1.8^b^
**Malp2**	90.6±2.2^b^	1.8±15.2	87.8±8.0^b^	−26.8±4.5^c^	31.7±19.6	97.5±3.1^b^
**Pam_3_**	91.5±0.4^b^	5.2±4.3	21.5±15.1	−22.0±1.9^c^	9.9±4.7	98.5±0.4^b^
**LPS**	7.2±27.7	−26.0±22.3^c^	−0.4±14.0^c^	99.3±0.9^b^	71.6±3.3^b^	55.4±6.0^b^

a BMDM from WT (C57Bl/6) or deficient mice (^−/−^) for TLR2, TLR1, TLR6, TLR4, CD14 and MyD88 primed with GM-CSF, were incubated with AraLAM (5000 ng/mL), LM (5000 ng/mL), Malp2 (300 ng/mL), Pam_3_-CSK_4_ (5000 ng/mL) or LPS (500 ng/mL) for 24 h *in vitro*. NO (Nitrite) was measured in the supernatants by Griess Method. Percentage of inhibition of NO production in deficient mice compared to WT.

b *p<0.05*, significantly versus WT production. Data are mean ± S.D, *n* = 6 mice per genotype from two independent experiments.

c Negative values corresponds an increment in correlation with WT production.

### 4. Regulation of mRNA Expression of the Enzymes Involved in PGE_2_ and LTB_4_ Metabolism, TLRs and TLR Adaptor Molecules in GM-CSF-primed BMDMs

**Figure 5:**
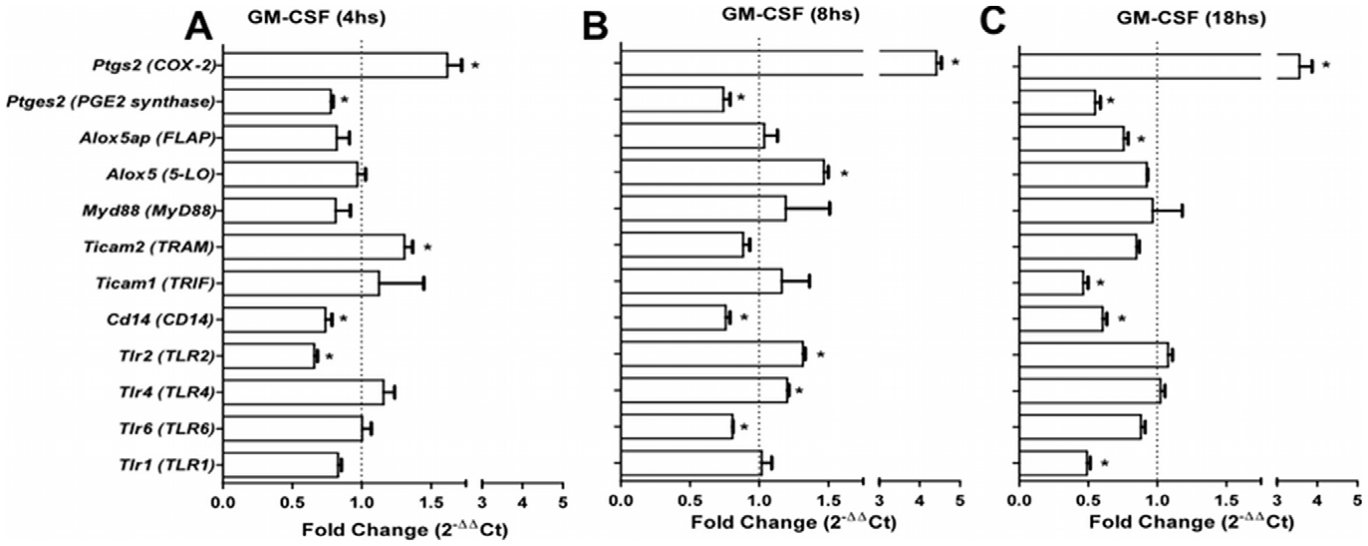
Effect of GM-CSF priming on BMDM mRNA expression. mRNA was prepared from 5×10^6^ BMDMs. Macrophages were treated with or without GM-CSF for 4 (**A**), 8 (**B**) or 18 h (**C**). qRT-PCR was used to determine the relative expression of transcripts encoding for lipid metabolism enzymes, TLRs and adaptor proteins. The results were normalized to the expression of the endogenous internal controls *Actb* and *Gapdh*. The 2–ΔΔCt method was used in the analysis of the qRT-PCR data. Dotted lines show mRNA expression in untreated cells. The results are presented as the average ± S.E.M from three independent experiments in duplicate (n = 3); *, *p*<0.05 compared with non-GM-CSF-treated cells.

To determine the effects of GM-CSF on steady-state mRNA encoding for 5-LO, FLAP, COX-2, PGE_2_ synthase, TLR1, TLR2, TLR4, TLR6, CD14, MyD88, TRIF and TRAM, macrophages were cultured with or without GM-CSF for various periods of time and without agonist stimulation. Quantitative real time polymerase chain reaction (qRT-PCR) analysis from these BMDMs revealed that GM-CSF priming increased COX*-2* mRNA expression (threefold) after 4 h of incubation (Fig. 5A), and this increase persisted through 8 h (fivefold) (Fig. 5B) and 18 h (fourfold) (Fig. 5C) of incubation. In contrast, PGE_2_ synthase mRNA was down-regulated 4 h after GM-CSF treatment and persisted until 18 h. No effect was observed on mRNA for either 5-LO or FLAP after 4 h of incubation, compared with non-treated macrophages (Fig. 5A). However, there was an approximately half-fold increase in mRNA for 5-LO but not for FLAP at 8 h of GM-CSF treatment (Fig. 5B). As shown in Fig. 5B, we detected increased mRNA expression of TLR2 and TLR4 only after 8 h of GM-CSF treatment. There was no effect on mRNA expression for TLR1 and TLR6. However, CD14 mRNA expression was down-regulated within 18 h of GM-CSF treatment. Since the use of different adaptor proteins provides a mechanism to modulate the response of individual TLRs, we analyzed the regulation of the expression of known TLR adaptor protein stimulation. TRAM mRNA expression is up-regulated after 4 h of treatment (Fig. 5A), which was associated with elevated MyD88 and TRIF mRNA expression after 8 h of GM-CSF treatment (Fig. 5B). Thus, GM-CSF coordinately regulates the expression of multiple molecular components of the innate response in macrophages, including both positive (COX-2, 5-LO, TLR2, TLR4, MyD88, TRIF and TRAM) and negative (PGE_2_ synthase and CD14) components.

### 5. TLRs and Co-receptors Participate in IL-10 Production by Macrophages Stimulated with Bacterial Products

**Figure 6:**
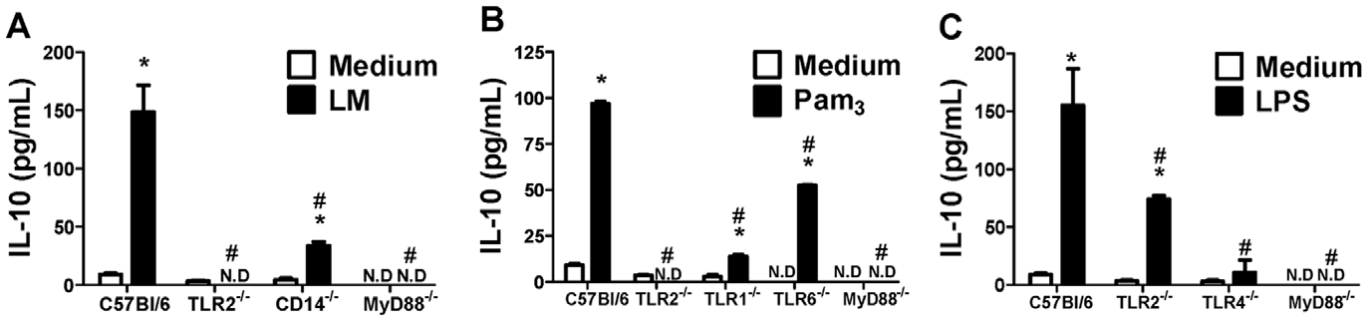
Evidence of the involvement of TLRs, CD14 and MyD88, in IL-10 production by BMDM primed with GM-CSF. BMDM from WT mice or mice deficient were primed with GM-CSF for 24 h and then stimulated with LM (5000 ng/mL), Pam_3_-CSK_4_ (5000 ng/mL) or LPS (500 ng/mL) for 24 h. The concentrations of IL-10 from BMDM deficient for TLR2, CD14 or MyD88 stimulated with LM (**B**), deficient for TLR2, TLR1, TLR6 and MyD88 stimulated with Pam_3_-CSK_4_ (**C**) and deficient for TLR2, TLR4 and MyD88 stimulated with LPS (**D**) were determined in the culture supernatant. Medium alone was used as a negative control. ELISA was used to determine the concentrations of IL-10. Results are the average ± S.E.M (*n* = 6 per genotype), from two independent experiments; *, *p*<*0.05* compared with unstimulated cells; **^#^**, *p<0.05* compared with WT mice.

In addition to their participation in the production of pro-inflammatory mediators, TLR-ligands are also involved in the regulation of IL-10 release, as shown in Figure 6. Macrophages primed with GM-CSF constitutively produced relatively low concentrations of IL-10. Stimulation with LM, Pam_3_-CSK_4_ and LPS resulted in IL-10 secretion. However, AraLAM and Malp2 had a very small effect on IL-10 induction (data not shown). Subsequently, the impact of TLRs and co-receptors on IL-10 production after GM-CSF priming was examined. As shown in Figure 6A, IL-10 secretion by LM-stimulated WT primary macrophages was inhibited in TLR2^−/−^, CD14^−/−^ and MyD88^−/−^ cells. Indeed, TLR2^−/−^, TLR1^−/−^ and MyD88^−/−^ cells were largely unresponsive to Pam_3_-CSK_4_ in terms of IL-10 release, and TLR6^−/−^ macrophages were partially responsive (Fig. 6B), while absence of TLR4 and MyD88 prevented LPS induced IL-10 release (Fig. 6C). Thus, bacterial PAMPs, including mycobacterial LM, induce IL-10 release in GM-CSF primed macrophages.

### 6. IFN-γ Potentiates the Effects of GM-CSF

**Figure 7:**
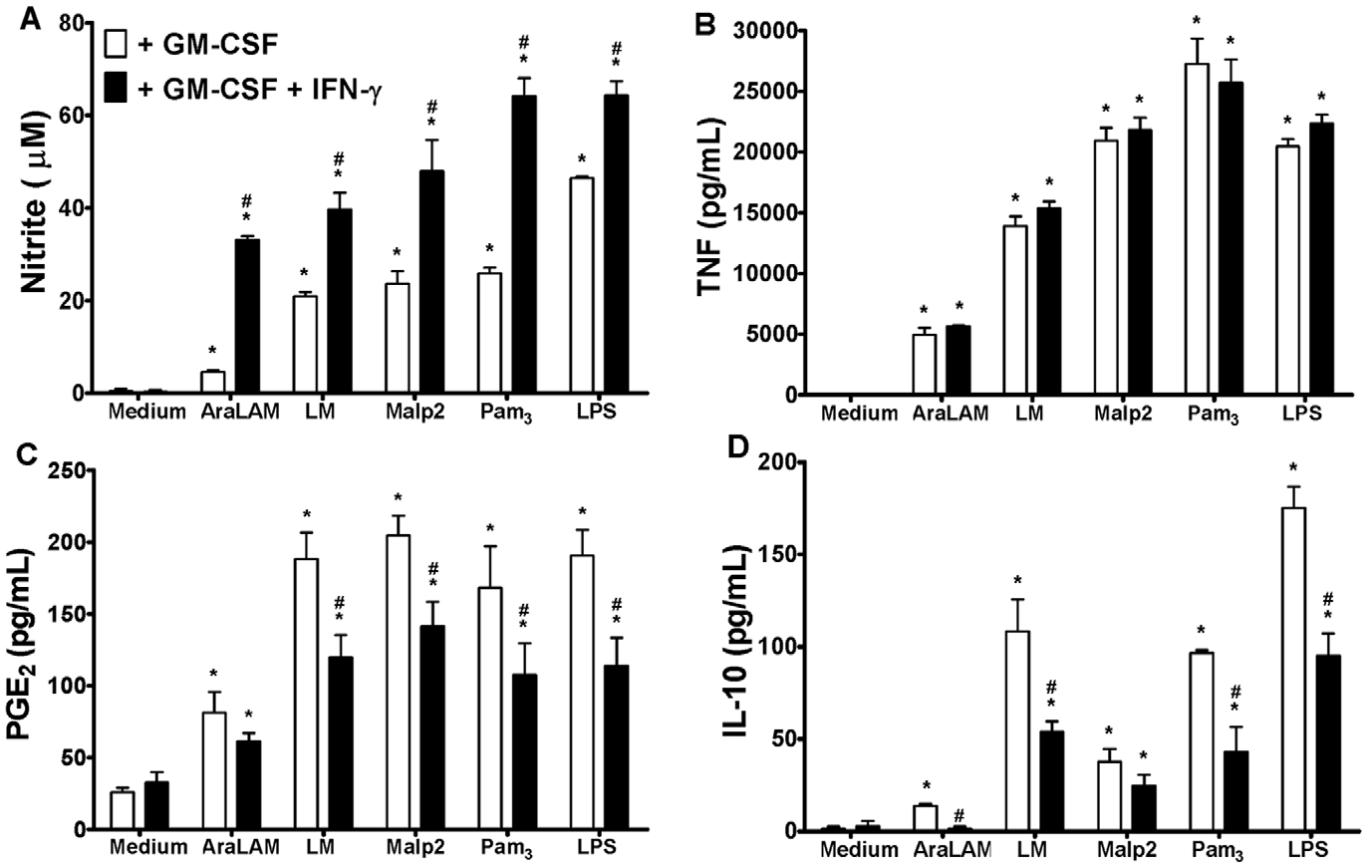
IFN-γ potentiates the GM-CSF priming effects on BMDM. BMDM WT cells were preincubated for 24 h with GM-CSF or GM-CSF plus IFN-γ (500 UI/mL) and then stimulated with AraLAM (5000 ng/mL), LM (5000 ng/mL), Malp2 (300 ng/mL), Pam_3_-CSK_4_ (5000 ng/mL) or LPS (500 ng/mL) for 24 h *in vitro*. The supernatants were analyzed for the production of NO (Nitrite) (**A**), TNF-α (**B**), PGE_2_ (**C**) and IL-10 (**D**). Medium alone was used as a negative control. ELISA was used to measure TNF-α and PGE_2_, and the Griess Method was used to measure Nitrite production in the supernatants. Results are the average ± S.E.M from mice cells (*n* = 6 per group) from two independent experiments; *, *p*<0.05 compared with unstimulated cells; **^#^**, *p<0.05* compared with GM-CSF priming alone.

IFN-γ is added in culture to prime macrophage. To analyze the effect of GM-CSF in combination with IFN-γ on macrophage priming, the production levels of TNF-α, IL-10, NO and PGE_2_ were determined in cell culture supernatants after stimulation with TLR ligands. Macrophages were primed with GM-CSF only or in combination with IFN-γ. For all TLRs ligands tests NO release was increased after priming with a combination of GM-CSF and IFN-γ (Fig. 7A). Priming with either GM-CSF or IFN-γ induced a comparable TNF-α release in response to the TLR ligands (data not shown). However, priming with the combination of GM-CSF plus IFN-γ did not result in a further increase in TNF-α release as compared to the single priming with GM-CSF, at the concentrations of stimuli used (Fig. 7B). Surprisingly, PGE_2_ (Fig. 7C) and IL-10 (Fig. 7D) production by macrophages primed with both GM-CSF and IFN-γ significantly decreased in response to AraLAM, LM, Malp2, Pam_3_-CSK_4_ or LPS exposure, compared to priming with GM-CSF alone. Thus, IFN-γ priming potentiates GM-CSF induced increase in NO and decrease in PGE_2_ and this is associated with a decrease in IL-10 release.

### 7. The Effects of GM-CSF Priming on NFκB Nuclear Translocation and PPAR-γ Activation Upon TLR2 Stimulation

**Figure 8:**
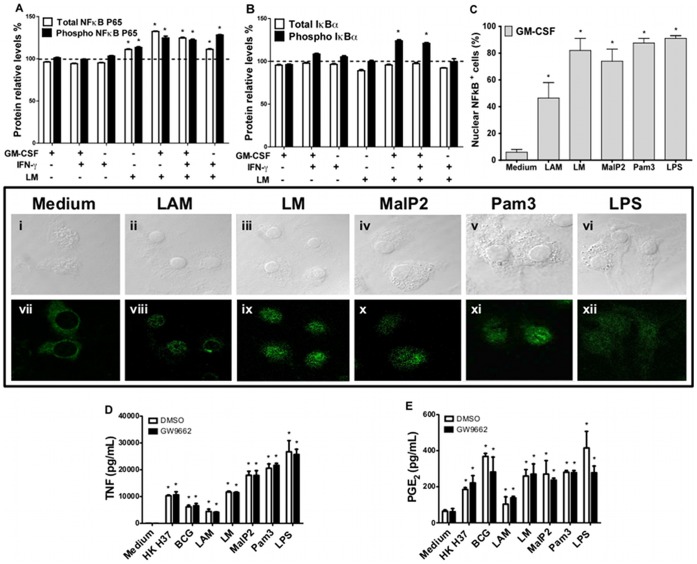
Nuclear translocation of NFκB and PPAR-γ activation in BMDMs primed with GM-CSF. NFκBp65, IκBα expression and NFκB nuclear translocation were investigated in BMDMs from WT mice. Graphic (**A**) percentage of relative expression of total NFκBp65, phosphorylated NFκBp65, (**B**) total IκBα and phosphorylated IκBα compared with non-stimulated control cell lysate (dotted line). Cells were pre-incubated for 24 h with GM-CSF, IFN-γ or GM-CSF plus IFN-γ and then stimulated with LM (5000 ng/mL) for 2 h *in vitro.* Total cellular proteins were collected to assay the relative expression by ELISA (PathScan Sandwich ELISA kit). The percentage of positive cells for NFkB nuclear translocation is shown for macrophages primed with GM-CSF for 24 h and then stimulated with different TLR2 agonists or LPS for 2 h, as analyzed by confocal microscopy. The **panel** depicts the expression of nuclear NFκB in GM-CSF primed BMDMs stimulated with medium (**vii**), AraLAM (**viii**), LM (**ix**), Malp2 (**x**), Pam_3_-CSK_4_ (**xi**) or LPS (**xii**) and the contrast DIC (i**, ii, iii, iv, v** and **vi**) on immunofluorescence slides. To evaluate the role of PPAR-γ in pro-inflammatory mediator release, WT primary macrophages were treated with 1 µM of PPAR-γ antagonist (GW 9662) 1 h before stimulus. TNF-α (**C**) and PGE_2_ production (**D**) after 24 h of incubation were measured directly in the supernatant by ELISA. The graphic results are the average ± S.E.M for cells (*n* = 6 per group) from two independent experiments; *, p<0.05 compared with unstimulated cells. The images from confocal microscopy were representatives from two independent experiments.

NFκB is an inducible transcription factor that plays a central role in the regulation of inflammation. In resting cells, NFκB is maintained in an inactive form in the cytoplasm by association with IκB, but after cell stimulation, IκB is phosphorylated, allowing NFκB to be translocated into the nucleus and to become active. To determine whether GM-CSF priming and stimulation with TLR2 ligands are associated with the activation of NFκB, we analyzed total cytoplasmic NFκBp65, phosphorylated (phospho) NFκBp65, total IκBα, and phospho-IκBα in BMDM lysate after priming the cells with GM-CSF, IFN-γ or GM-CSF plus IFN-γ in the presence or absence of LM stimulus (2 h) using PathScan® ELISA. The results showed that all priming had no effects on total and phospho-NFκBp65 expression (Fig. 8A) or total and phospho-IκBα expression (Fig. 8B). The LM stimulus up-regulated total and phospho-NFκBp65 but had no effect on total and phospho-IκBα expression. Priming with GM-CSF or GM-CSF plus IFN-γ enhanced the total and phospho-NFκBp65 expression after LM stimulation. Indeed, these priming conditions also enhanced phospho-IκBα expression. However, the priming with only IFN-γ increased phospho-NFκBp65 but not phospho-IκBα expression after LM stimulation. The nuclear translocation of NFκBp65 was confirmed by immunofluorescence. NFκBp65 staining is cytoplasmic in GM-CSF-primed macrophages before TLR ligand stimulation. After 2 h of stimulation with AraLAM, LM, Malp2, Pam3-CSK4 or LPS, the translocation to the nucleus was clear (Fig. 8, panel). The stimuli also significantly increased the percentage of cells showing nuclear NFκBp65 staining, as compared with the untreated cells (Fig. 8C).

Considering that lipid-activated nuclear receptors may play an important role in macrophage differentiation and in the inflammatory response, we next examined the influence of PPAR-γ inhibition on TNF-α and PGE_2_ production by TLR-stimulated macrophages primed with GM-CSF using a specific PPAR-γ antagonist (GW9662). Primed macrophages were pre-treated with GW9662 and then stimulated with AraLAM, LM, Malp2, Pam3-CSK4, and LPS, using heat-killed *M. tuberculosis* H37Rv (HK H37) and *M. bovis* BCG as positive controls. Pre-treatment with the PPAR-γ antagonist GW9662 had no effect on the production of TNF-α (Fig. 8C) and PGE_2_ (Fig. 8D) in primed macrophages after stimulation. Therefore, NFκB, but not PPAR-γ, seems to be involved in the GM-CSF-primed macrophage response to TLR2 and TLR4 agonists, resulting in TNF-α, NO and PGE_2_ release.

## Discussion

Macrophages have a pivotal function in the resolution of infectious diseases. The activation of macrophage receptors induces intracellular signaling that results in the release of mediators into the microenvironment. GM-CSF is a multifunctional cytokine that has a primary role as a hematopoietic growth factor. In addition, GM-CSF can directly activate and have a priming effect on mature phagocytes [42], [43]. TLRs interact with different combinations of adapter proteins and activate transcription factors, leading to specific immune responses when activated by structurally conserved molecules derived from pathogens [44], [45]. Here, we analyzed the mechanisms governing TLR-mediated eicosanoid production by BMDMs primed with GM-CSF. Primarily, our study demonstrated that priming BMDMs with GM-CSF and stimulating BMDMs with TLR ligands results in a downregulation of PGE_2_ production. In contrast, these primed cells have increased TNF-α and NO release. The production of pro-inflammatory mediators by GM-CSF-primed BMDMs is dependent on the adaptor protein MyD88 and the nuclear translocation of NFκBp65.

As an activator of cell functions, GM-CSF has been shown to upregulate LTs release by alveolar macrophages (AM) in rats [9]. When pre-treated with GM-CSF, AMs released more AA and generated more metabolites, including PGs and LTs upon subsequent stimulation, compared to untreated cells. This action was related to the capacity of GM-CSF to increase the expression of the 85-kDa PLA2 protein, an important enzyme in the eicosanoid production pathway [9]. Here, priming with GM-CSF resulted in a reduction of PGE_2_ release by BMDMs stimulated with different TLR2 ligands (AraLAM, LM, Malp2 and Pam_3_-CSK_4_) or the TLR4 ligand LPS. Although this reduction was accompanied by an increase in COX-2 mRNA expression, PGE-synthase mRNA expression was downregulated at the time of the analyses, and this downregulation may have led to the decrease in PGE_2_ production. Several reports demonstrated that PGE_2_ contributes to immune suppression [46]. PGE_2_ secreted from activated macrophages in response to pro-inflammatory stimuli acts on macrophages themselves and exhibits an inhibitory function in a negative feedback loop [47], [48]. Therefore, we suggest that GM-CSF acted as a regulator for an inflammatory profile. The biosynthesis of LTs in macrophages is known to result from a complex series of reactions. These particular events begin with the activation of transmembrane receptors by cytokines or other stimuli that prompt the activation and expression of 5-LO and the entire signaling pathway, resulting in the production of LTB_4_ and cysteinyl leukotrienes (Cys-LT). Here, although the effect of GM-CSF priming on BMDM release of LTB_4_ was demonstrated and 5-LO mRNA expression was upregulated in BMDMs after 8 h of GM-CSF priming, no further increase in LTB_4_ was observed after stimulation with TLR2 ligands or LPS. These results suggest that there is additional regulation at other levels in the 5-LO pathway and may reflect the activation/deactivation of the cell signaling response to a stimulatory assay [49].

Priming with GM-CSF was also critical for the formation of NO by BMDMs. In GM-CSF-primed macrophages, the level of NO production was strongly increased with all the stimuli evaluated. The increase in NO production may contribute to the decrease in PGE_2_ release. NO has been reported to suppress COX activity and PGE_2_ production [50]. The modulation of the COX system by NO represents an important pathway that regulates the magnitude of the inflammatory process [51]. There is molecular crosstalk between the inflammatory mediators NO and PGs. NO exerts divergent effects on the constitutive and inducible COX isoforms, activating COX-1 but inactivating COX-2 [52]. Moreover, COX-2 nitration inhibits the catalytic activity of this enzyme [53]. Interestingly, other reports suggest that LPS reduces 5-LO metabolism by multiple mechanisms, including the marked induction of NO synthase (NOS) [54]. NO has been proposed to inactivate lipoxygenases by reducing the ferric enzyme to the ferrous form [55]. This effect of NO on 5-LO and COX-2 inactivation is another possible mechanism for the lower production of LTB_4_ and the decreased PGE_2_ production by GM-CSF-primed BMDMs after TLR-ligand stimulation.

Different from that observed with PGE_2_, GM-CSF-primed BMDMs subsequently stimulated with TLR ligands produced higher concentrations of TNF-α, as compared with unprimed cells. These data confirm the results of a study demonstrating that the main priming factor for TNF-α release by human PMNs was GM-CSF [56]. We suggest that the increase in cytokines release after priming with GM-CSF could be a consequence of a series of events, such as an increase in TLR2 and TLR4 mRNA expression and an increase in mRNA expression of TRIF-related adaptor molecule (TRAM) and MyD88. However, IL-10 production in response to LM, Pam_3_-CSK_4_ or LPS was reduced in GM-CSF-primed macrophages (data not shown). Our results demonstrate that GM-CSF priming enhances phospho-IκBα expression after TLR2 agonist stimulation and that IFN-γ potentiates this phenomenon. Increased IκBα phosphorylation and elevated levels of nuclear NFκB components p50 and p65 by GM-CSF-priming was also demonstrated in monocytes [43].

We addressed the effect of GM-CSF priming in combination with IFN-γ in macrophage stimulation. IFN-γ is a major activation signal for macrophages. The priming or concomitant exposure of macrophages to IFN-γ dramatically increases their responsiveness to microbial products, such as LPS, in terms of the production of NO, cytokines and co-stimulatory molecules [57]. Macrophage priming with the combination of GM-CSF and IFN-γ had no effect on TNF-α release, possibly because priming with GM-CSF alone was able to induce the maximal production of this cytokine at the doses of agonists that were used. However, NO production was increased in the presence of IFN-γ, demonstrating its importance on cell signaling to activate NO metabolism in macrophages. As expected, with increased concentrations of NO, there was decreased PGE_2_ and IL-10 production by BMDMs primed with the combination of GM-CSF and IFN-γ, in comparison to BMDMs primed with GM-CSF alone. Indeed, GM-CSF and IFN-γ have immunostimulatory properties; they not only prime resting monocytes but also restore monocytic functions after LPS tolerization [58].

The concept of TLR-ligand interactions suggests that individual TLR proteins recognize a set of microbial products [40]. Some TLR proteins may act cooperatively in response to particular microbial ligands [38]. In studies of neutrophil priming, the expression of TLR2 and CD14 was upregulated by GM-CSF or LPS treatment [4]. Our results showed that TLR2 and TLR4 expression by BMDMs controlled the potential response to microbial ligands and that this response was dramatically enhanced by GM-CSF priming, suggesting that GM-CSF can modulate the activation of macrophages in the host defense against bacterial infection. The mycobacterial lipoglycans LM and AraLAM are structural parts of the mycobacterial cell wall that interact with pattern recognition receptors, such as TLRs [19]. AraLAM and LM from several mycobacteria display pro-inflammatory activities [59], [60], whereas ManLAM has a predominant anti-inflammatory activity in the presence of the TLR4 agonist LPS [61], [62]. In our experiments, we used LM and AraLAM purified from *M. smegmatis*, and we described the pattern of recognition of GM-CSF-primed BMDMs by TLR2, TLR1, TLR6, TLR4 and CD14 agonists. The release of TNF-α from AraLAM-stimulated cells in culture is mediated through TLR2 and CD14. However, NO production and PGE_2_ production are mediated by only TLR2. As we demonstrated here, LM stimulation of PGE_2_ formation occurs by activating a complex cluster of receptors mediated through CD14, TLR2 and TLR1, which may function as heterodimers. The lipopeptides induce signaling in the cells of the immune system through TLR2–TLR1 or TLR2–TLR6 heterodimers [63], [64]. We show that Malp2 stimulation of GM-CSF-primed macrophages results in a marked increase in pro-inflammatory mediator production. Malp2 stimulation of PGE_2_ release is strongly dependent on TLR2–TLR6 and the adaptor protein MyD88 in GM-CSF-primed macrophages, while Pam_3_-CSK_4_ stimulation of PGE_2_ release depends mostly on the TLR2 and MyD88 pathway.

NFκB plays a central role in the regulation of many of the genes responsible for the generation of inflammatory mediators. [65]. Therefore, NFκB is an attractive candidate to mediate the release of pro-inflammatory mediators. Our data show that BMDMs primed with GM-CSF and stimulated with TLR2 ligands express a very prominent nuclear staining of NFκBp65 and phospho-NFκBp65 relative expression. Peroxisome proliferator activated receptors (PPARs) are members of the nuclear hormone receptor superfamily of ligand-activated transcription factors [66]. Mycobacteria-induced PPAR-γ expression and activation are demonstrated to be centrally involved in regulating lipid metabolism in macrophages through the modulation of lipid body biogenesis and PGE_2_ production, thereby affecting the host response to infection [67]. We hypothesized that the activation of PPAR-γ has an effect on PGE_2_ release by macrophages. However, PPAR-γ inhibition by GW9662 treatment did not affect TNF-α and PGE_2_ release in response to TLR agonists, heat-killed *M. tuberculosis*, or BCG infection.

Together, our results demonstrate that GM-CSF priming of BMDMs enhances the capacity to drive the pro-inflammatory response against bacterial components by increasing TNF-α and NO production and by altering lipid mediator release. In particular, the production of PGE_2_, which was more prominent than that of LTB_4_, was strongly decreased by GM-CSF priming, indicating a compensatory function for these cells in a high inflammatory environment. Our data suggest that the GM-CSF produced when a host is infected by a pathogen may be responsible for the macrophages exhibiting a pro-inflammatory profile. Additionally, GM-CSF drives host immune defense mechanisms, which favor phagocytosis and killing, as a result of a shift in the production of lipid mediators from PGE_2_ to LTB_4._

## References

[1] Pluddemann A, Mukhopadhyay S, Gordon S (2011). Innate immunity to intracellular pathogens: macrophage receptors and responses to microbial entry.. Immunol Rev.

[2] Marim FM, Silveira TN, Lima DS, Zamboni DS (2010). A method for generation of bone marrow-derived macrophages from cryopreserved mouse bone marrow cells.. PLoS One.

[3] Peters-Golden M, McNish RW, Hyzy R, Shelly C, Toews GB (1990). Alterations in the pattern of arachidonate metabolism accompany rat macrophage differentiation in the lung.. Journal of immunology.

[4] Kurt-Jones EA, Mandell L, Whitney C, Padgett A, Gosselin K (2002). Role of toll-like receptor 2 (TLR2) in neutrophil activation: GM-CSF enhances TLR2 expression and TLR2-mediated interleukin 8 responses in neutrophils.. Blood.

[5] DiPersio JF, Billing P, Williams R, Gasson JC (1988). Human granulocyte-macrophage colony-stimulating factor and other cytokines prime human neutrophils for enhanced arachidonic acid release and leukotriene B4 synthesis.. Journal of immunology.

[6] McColl SR, Krump E, Naccache PH, Poubelle PE, Braquet P (1991). Granulocyte-macrophage colony-stimulating factor increases the synthesis of leukotriene B4 by human neutrophils in response to platelet-activating factor. Enhancement of both arachidonic acid availability and 5-lipoxygenase activation.. Journal of immunology.

[7] Krump E, Borgeat P (1994). Kinetics of 5-lipoxygenase activation, arachidonic acid release, and leukotriene synthesis in human neutrophils: effects of granulocyte-macrophage colony-stimulating factor.. Biochimica et biophysica acta.

[8] Pouliot M, McDonald PP, Khamzina L, Borgeat P, McColl SR (1994). Granulocyte-macrophage colony-stimulating factor enhances 5-lipoxygenase levels in human polymorphonuclear leukocytes.. Journal of immunology.

[9] Brock TG, McNish RW, Coffey MJ, Ojo TC, Phare SM (1996). Effects of granulocyte-macrophage colony-stimulating factor on eicosanoid production by mononuclear phagocytes.. Journal of immunology.

[10] Paine R, 3rd, Preston AM, Wilcoxen S, Jin H, Siu BB, et al (2000). Granulocyte-macrophage colony-stimulating factor in the innate immune response to Pneumocystis carinii pneumonia in mice.. J Immunol.

[11] Ballinger MN, Paine R, 3rd, Serezani CH, Aronoff DM, Choi ES, et al (2006). Role of granulocyte macrophage colony-stimulating factor during gram-negative lung infection with Pseudomonas aeruginosa.. American journal of respiratory cell and molecular biology.

[12] Goldstein E, Lippert W, Warshauer D (1974). Pulmonary alveolar macrophage. Defender against bacterial infection of the lung.. J Clin Invest.

[13] Berclaz PY, Zsengeller Z, Shibata Y, Otake K, Strasbaugh S (2002). Endocytic internalization of adenovirus, nonspecific phagocytosis, and cytoskeletal organization are coordinately regulated in alveolar macrophages by GM-CSF and PU.1.. J Immunol.

[14] Berclaz PY, Shibata Y, Whitsett JA, Trapnell BC (2002). GM-CSF, via PU.1, regulates alveolar macrophage Fcgamma R-mediated phagocytosis and the IL-18/IFN-gamma -mediated molecular connection between innate and adaptive immunity in the lung.. Blood.

[15] Brightbill HD, Libraty DH, Krutzik SR, Yang RB, Belisle JT (1999). Host defense mechanisms triggered by microbial lipoproteins through toll-like receptors.. Science.

[16] Lien E, Sellati TJ, Yoshimura A, Flo TH, Rawadi G (1999). Toll-like receptor 2 functions as a pattern recognition receptor for diverse bacterial products.. The Journal of biological chemistry.

[17] Takeuchi O, Hoshino K, Kawai T, Sanjo H, Takada H (1999). Differential roles of TLR2 and TLR4 in recognition of gram-negative and gram-positive bacterial cell wall components.. Immunity.

[18] Campos MA, Almeida IC, Takeuchi O, Akira S, Valente EP (2001). Activation of Toll-like receptor-2 by glycosylphosphatidylinositol anchors from a protozoan parasite.. Journal of immunology.

[19] Means TK, Lien E, Yoshimura A, Wang S, Golenbock DT (1999). The CD14 ligands lipoarabinomannan and lipopolysaccharide differ in their requirement for Toll-like receptors.. Journal of immunology.

[20] Takeuchi O, Sato S, Horiuchi T, Hoshino K, Takeda K (2002). Cutting edge: role of Toll-like receptor 1 in mediating immune response to microbial lipoproteins.. Journal of immunology.

[21] Aderem A, Ulevitch RJ (2000). Toll-like receptors in the induction of the innate immune response.. Nature.

[22] Sheedy FJ, O’Neill LA (2007). The Troll in Toll: Mal and Tram as bridges for TLR2 and TLR4 signaling.. Journal of leukocyte biology.

[23] Peters-Golden M, Henderson WR (2007). Leukotrienes.. The New England journal of medicine.

[24] Flower RJ (2006). Prostaglandins, bioassay and inflammation.. British journal of pharmacology.

[25] Peters-Golden M, Canetti C, Mancuso P, Coffey MJ (2005). Leukotrienes: underappreciated mediators of innate immune responses.. Journal of immunology.

[26] Peres CM, de Paula L, Medeiros AI, Sorgi CA, Soares EG (2007). Inhibition of leukotriene biosynthesis abrogates the host control of Mycobacterium tuberculosis.. Microbes Infect.

[27] Medeiros AI, Sa-Nunes A, Soares EG, Peres CM, Silva CL (2004). Blockade of endogenous leukotrienes exacerbates pulmonary histoplasmosis.. Infect Immun.

[28] Aronoff DM, Peres CM, Serezani CH, Ballinger MN, Carstens JK (2007). Synthetic prostacyclin analogs differentially regulate macrophage function via distinct analog-receptor binding specificities.. Journal of immunology.

[29] Machado ER, Carlos D, Lourenco EV, Souza GE, Sorgi CA (2010). Cyclooxygenase-derived mediators regulate the immunological control of Strongyloides venezuelensis infection.. FEMS Immunol Med Microbiol.

[30] Medeiros AI, Serezani CH, Lee SP, Peters-Golden M (2009). Efferocytosis impairs pulmonary macrophage and lung antibacterial function via PGE2/EP2 signaling.. J Exp Med.

[31] Takeuchi O, Sato S, Horiuchi T, Hoshino K, Takeda K (2002). Cutting edge: role of Toll-like receptor 1 in mediating immune response to microbial lipoproteins.. J Immunol.

[32] Takeuchi O, Kawai T, Muhlradt PF, Morr M, Radolf JD (2001). Discrimination of bacterial lipoproteins by Toll-like receptor 6.. Int Immunol.

[33] Yang KK, Dorner BG, Merkel U, Ryffel B, Schutt C (2002). Neutrophil influx in response to a peritoneal infection with Salmonella is delayed in lipopolysaccharide-binding protein or CD14-deficient mice.. J Immunol.

[34] Kawai T, Adachi O, Ogawa T, Takeda K, Akira S (1999). Unresponsiveness of MyD88-deficient mice to endotoxin.. Immunity.

[35] Hoshino K, Takeuchi O, Kawai T, Sanjo H, Ogawa T (1999). Cutting edge: Toll-like receptor 4 (TLR4)-deficient mice are hyporesponsive to lipopolysaccharide: evidence for TLR4 as the Lps gene product.. J Immunol.

[36] Green LC, Wagner DA, Glogowski J, Skipper PL, Wishnok JS (1982). Analysis of nitrate, nitrite, and [15N]nitrate in biological fluids.. Analytical biochemistry.

[37] Doz E, Rose S, Court N, Front S, Vasseur V (2009). Mycobacterial phosphatidylinositol mannosides negatively regulate host Toll-like receptor 4, MyD88-dependent proinflammatory cytokines, and TRIF-dependent co-stimulatory molecule expression.. J Biol Chem.

[38] Ozinsky A, Underhill DM, Fontenot JD, Hajjar AM, Smith KD (2000). The repertoire for pattern recognition of pathogens by the innate immune system is defined by cooperation between toll-like receptors.. Proceedings of the National Academy of Sciences of the United States of America.

[39] Henneke P, Takeuchi O, van Strijp JA, Guttormsen HK, Smith JA (2001). Novel engagement of CD14 and multiple toll-like receptors by group B streptococci.. Journal of immunology.

[40] Akira S, Takeda K, Kaisho T (2001). Toll-like receptors: critical proteins linking innate and acquired immunity.. Nature immunology.

[41] Dunne A, O’Neill LA (2005). Adaptor usage and Toll-like receptor signaling specificity.. FEBS letters.

[42] Gasson JC, Weisbart RH, Kaufman SE, Clark SC, Hewick RM (1984). Purified human granulocyte-macrophage colony-stimulating factor: direct action on neutrophils.. Science.

[43] Lendemans S, Rani M, Selbach C, Kreuzfelder E, Schade FU (2006). GM-CSF priming of human monocytes is dependent on ERK1/2 activation.. Journal of endotoxin research.

[44] Medzhitov R (2007). Recognition of microorganisms and activation of the immune response.. Nature.

[45] Takeda K, Kaisho T, Akira S (2003). Toll-like receptors.. Annual review of immunology.

[46] Aronoff DM, Canetti C, Peters-Golden M (2004). Prostaglandin E2 inhibits alveolar macrophage phagocytosis through an E-prostanoid 2 receptor-mediated increase in intracellular cyclic AMP.. J Immunol.

[47] Ikegami R, Sugimoto Y, Segi E, Katsuyama M, Karahashi H (2001). The expression of prostaglandin E receptors EP2 and EP4 and their different regulation by lipopolysaccharide in C3H/HeN peritoneal macrophages.. Journal of immunology.

[48] Aronoff DM, Peres CM, Serezani CH, Ballinger MN, Carstens JK (2007). Synthetic prostacyclin analogs differentially regulate macrophage function via distinct analog-receptor binding specificities.. J Immunol.

[49] Radmark O, Werz O, Steinhilber D, Samuelsson B (2007). 5-Lipoxygenase: regulation of expression and enzyme activity.. Trends in biochemical sciences.

[50] Stadler J, Harbrecht BG, Di Silvio M, Curran RD, Jordan ML (1993). Endogenous nitric oxide inhibits the synthesis of cyclooxygenase products and interleukin-6 by rat Kupffer cells.. Journal of leukocyte biology.

[51] Vane JR, Mitchell JA, Appleton I, Tomlinson A, Bishop-Bailey D (1994). Inducible isoforms of cyclooxygenase and nitric-oxide synthase in inflammation.. Proceedings of the National Academy of Sciences of the United States of America.

[52] Clancy R, Varenika B, Huang W, Ballou L, Attur M (2000). Nitric oxide synthase/COX cross-talk: nitric oxide activates COX-1 but inhibits COX-2-derived prostaglandin production.. Journal of immunology.

[53] Goodwin DC, Gunther MR, Hsi LC, Crews BC, Eling TE (1998). Nitric oxide trapping of tyrosyl radicals generated during prostaglandin endoperoxide synthase turnover. Detection of the radical derivative of tyrosine 385.. The Journal of biological chemistry.

[54] Coffey MJ, Phare SM, Peters-Golden M (2000). Prolonged exposure to lipopolysaccharide inhibits macrophage 5-lipoxygenase metabolism via induction of nitric oxide synthesis.. Journal of immunology.

[55] Maccarrone M, Corasaniti MT, Guerrieri P, Nistico G, Finazzi Agro A (1996). Nitric oxide-donor compounds inhibit lipoxygenase activity.. Biochemical and biophysical research communications.

[56] Jablonska E, Kiluk M, Markiewicz W, Jablonski J (2002). Priming effects of GM-CSF, IFN-gamma and TNF-alpha on human neutrophil inflammatory cytokine production.. Melanoma research.

[57] Nathan CF, Prendergast TJ, Wiebe ME, Stanley ER, Platzer E (1984). Activation of human macrophages. Comparison of other cytokines with interferon-gamma.. The Journal of experimental medicine.

[58] Randow F, Docke WD, Bundschuh DS, Hartung T, Wendel A (1997). In vitro prevention and reversal of lipopolysaccharide desensitization by IFN-gamma, IL-12, and granulocyte-macrophage colony-stimulating factor.. Journal of immunology.

[59] Vignal C, Guerardel Y, Kremer L, Masson M, Legrand D (2003). Lipomannans, but not lipoarabinomannans, purified from Mycobacterium chelonae and Mycobacterium kansasii induce TNF-alpha and IL-8 secretion by a CD14-toll-like receptor 2-dependent mechanism.. J Immunol.

[60] Quesniaux VJ, Nicolle DM, Torres D, Kremer L, Guerardel Y (2004). Toll-like receptor 2 (TLR2)-dependent-positive and TLR2-independent-negative regulation of proinflammatory cytokines by mycobacterial lipomannans.. J Immunol.

[61] Geijtenbeek TB, Van Vliet SJ, Koppel EA, Sanchez-Hernandez M, Vandenbroucke-Grauls CM (2003). Mycobacteria target DC-SIGN to suppress dendritic cell function.. J Exp Med.

[62] Nigou J, Zelle-Rieser C, Gilleron M, Thurnher M, Puzo G (2001). Mannosylated lipoarabinomannans inhibit IL-12 production by human dendritic cells: evidence for a negative signal delivered through the mannose receptor.. J Immunol.

[63] Morr M, Takeuchi O, Akira S, Simon MM, Muhlradt PF (2002). Differential recognition of structural details of bacterial lipopeptides by toll-like receptors.. European journal of immunology.

[64] Takeuchi O, Kaufmann A, Grote K, Kawai T, Hoshino K (2000). Cutting edge: preferentially the R-stereoisomer of the mycoplasmal lipopeptide macrophage-activating lipopeptide-2 activates immune cells through a toll-like receptor 2- and MyD88-dependent signaling pathway.. Journal of immunology.

[65] Peters RT, Liao SM, Maniatis T (2000). IKKepsilon is part of a novel PMA-inducible IkappaB kinase complex.. Molecular cell.

[66] Evans RM (1988). The steroid and thyroid hormone receptor superfamily.. Science.

[67] Almeida PE, Silva AR, Maya-Monteiro CM, Torocsik D, D’Avila H (2009). Mycobacterium bovis bacillus Calmette-Guerin infection induces TLR2-dependent peroxisome proliferator-activated receptor gamma expression and activation: functions in inflammation, lipid metabolism, and pathogenesis.. Journal of immunology.

